# Predicting the Occurrence of Variants in *RAG1* and *RAG2*

**DOI:** 10.1007/s10875-019-00670-z

**Published:** 2019-08-06

**Authors:** Dylan Lawless, Hana Lango Allen, James Thaventhiran, NIHR BioResource–Rare Diseases Consortium, Flavia Hodel, Rashida Anwar, Jacques Fellay, Jolan E. Walter, Sinisa Savic

**Affiliations:** 1 Leeds Institute of Biomedical and Clinical Sciences, St James’s University Hospital, University of Leeds, Wellcome Trust Brenner Building, Beckett Street, Leeds, UK; 2 Global Health Institute, School of Life Sciences, École Polytechnique Fédérale de Lausanne, 1015 Lausanne, Switzerland; 3 NIHR BioResource, Cambridge University Hospitals, Cambridge Biomedical Campus, Cambridge, CB20QQ UK; 4 Department of Haematology, University of Cambridge, Cambridge Biomedical Campus, Cambridge, CB20XY UK; 5 MRC Toxicology Unit, School of Biological Sciences, University of Cambridge, Cambridge, UK; 6 Swiss Institute of Bioinformatics, 1015 Lausanne, Switzerland; 7 University of South Florida and Johns Hopkins All Children’s Hospital, Saint Petersburg, FL USA; 8 Division of Allergy Immunology, Massachusetts General Hospital for Children, Boston, MA USA; 9 Department of Clinical Immunology and Allergy, St James’s University Hospital, Beckett Street, Leeds, UK; 10 National Institute for Health Research Leeds Musculoskeletal Biomedical Research Centre and Leeds Institute of Rheumatic and Musculoskeletal Medicine, St James’s University Hospital, Wellcome Trust Brenner Building, Beckett Street, Leeds, UK

**Keywords:** Recombination activating genes 1 and 2 (RAG1, RAG2), pathogenic variant, genomics, predictive

## Abstract

While widespread genome sequencing ushers in a new era of preventive medicine, the tools for predictive genomics are still lacking. Time and resource limitations mean that human diseases remain uncharacterized because of an inability to predict clinically relevant genetic variants. A strategy of targeting highly conserved protein regions is used commonly in functional studies. However, this benefit is lost for rare diseases where the attributable genes are mostly conserved. An immunological disorder exemplifying this challenge occurs through damaging mutations in *RAG1* and *RAG2* which presents at an early age with a distinct phenotype of life-threatening immunodeficiency or autoimmunity. Many tools exist for variant pathogenicity prediction, but these cannot account for the probability of variant occurrence. Here, we present a method that predicts the likelihood of mutation for every amino acid residue in the RAG1 and RAG2 proteins. Population genetics data from approximately 146,000 individuals was used for rare variant analysis. Forty-four known pathogenic variants reported in patients and recombination activity measurements from 110 RAG1/2 mutants were used to validate calculated scores. Probabilities were compared with 98 currently known human cases of disease. A genome sequence dataset of 558 patients who have primary immunodeficiency but that are negative for RAG deficiency were also used as validation controls. We compared the difference between mutation likelihood and pathogenicity prediction. Our method builds a map of most probable mutations allowing pre-emptive functional analysis. This method may be applied to other diseases with hopes of improving preparedness for clinical diagnosis.

**Electronic supplementary material:**

The online version of this article (10.1007/s10875-019-00670-z) contains supplementary material, which is available to authorized users.

## Introduction

Costs associated with genomic investigations continue to reduce [1], while the richness of data generated increases. Globally, the adoption of wide-scale genome sequencing implies that all newborn infants may receive screening for pathogenic genetic variants in an asymptomatic stage, pre-emptively [2]. The one dimensionality of individual genomes is now being expanded by the possibility of massive parallel sequencing for somatic variant analysis and by single-cell or lineage-specific genotyping, culminating in a genotype spectrum. In whole blood, virtually every nucleotide position may be mutated across 10^5^ cells [3]. Mapping one’s genotype across multiple cell types and at several periods during a person’s life may soon be feasible [4]. Such genotype snapshots might allow for prediction and tracking of somatic, epigenetic, and transcriptomic profiling.

The predictive value of genomic screening highly depends on the computation tools used for data analysis and its correlation with functional assays or prior clinical experience. Interpretation of that data is especially challenging for rare human genetic disorders; candidate disease-causing variants that are predicted as pathogenic often require complex functional investigations to confirm their significance. There is a need for predictive genomic modelling with aims to provide reliable guidance for therapeutic intervention for patients harboring genetic defects for life-threatening disease before the illness becomes clinically significant.

The study of predictive genomics is exemplified by consideration of gene essentiality, accomplished by observing intolerance to loss-of-function variants. Several gene essentiality scoring methods are available for both the coding and non-coding genome [5]. Approximately 3000 human genes cannot tolerate the loss of one allele [5]. The greatest hurdle in monogenic disease is the interpretation of variants of unknown significance while functional validation is a major time and cost investment for laboratories investigating rare disease.

Severe, life-threatening immune diseases are caused by genetic variations in almost 300 genes [6, 7]; however, only a small percentage of disease-causing variants have been characterized using functional studies. Several robust tools are in common usage for predicting variant pathogenicity. Compared with methods for pathogenicity prediction, a void remains for predicting mutation probability, essential for efficient pre-emptive validation. Our investigation aims to apply predictive genomics as a tool to identify genetic variants that are most likely to be seen in patient cohorts.

We present the first application of our novel approach of predictive genomics using Recombination activating gene 1 (RAG1) and RAG2 deficiency as a model for a rare primary immunodeficiency (PID) caused by autosomal recessive variants. *RAG1* and *RAG2* encode lymphoid-specific proteins that are essential for V(D)J recombination. This genetic recombination mechanism is essential for a robust immune response by diversification of the T and B cell repertoire in the thymus and bone marrow, respectively [8, 9]. Deficiency of RAG1 [10] and RAG2 [11] in mice causes inhibition of B and T cell development. Schwarz et al. [12] formed the first publication reporting that RAG mutations in humans cause severe combined immunodeficiency (SCID), and deficiency in peripheral B and T cells. Patient studies identified a form of immune dysregulation known as Omenn syndrome [13, 14]. The patient phenotype includes multi-organ infiltration with oligoclonal, activated T cells. The first reported cases of Omenn syndrome identified infants with hypomorphic RAG variants which retained partial recombination activity [15]. RAG deficiency can be measured by in vitro quantification of recombination activity [16–18]. Hypomorphic *RAG1* and *RAG2* mutations, responsible for residual V(D)J recombination activity (on average 5–30%), result in a distinct phenotype of combined immunodeficiency with granuloma and/or autoimmunity (CID-G/A) [2, 19, 20].

Human RAG deficiency has traditionally been identified at very early ages due to the rapid drop of maternally acquired antibody in the first six months of life. A loss of adequate lymphocyte development quickly results in compromised immune responses. More recently, we have found that RAG deficiency is also found for some adults living with PID [16].

*RAG1* and *RAG2* are highly conserved genes, but disease is only reported with autosomal recessive inheritance. Only 44% of amino acids in RAG1 and RAG2 are reported as mutated on GnomAD, and functional validation of candidate variants is difficult [21]. Pre-emptive selection of residues for functional validation is a major challenge; a selection based on low allele frequency alone is infeasible since the majority of each gene is highly conserved. A shortened time between genetic analysis and diagnosis means that treatments may be delivered earlier. RAG deficiency may present with diverse phenotypes, and treatment strategies vary. With such tools, early intervention may be prompted. Some patients could benefit from hematopoietic stem cell transplant [22] when necessary, while others may be provided mechanism-based treatment [23]. Here, we provide a new method for predictive scoring that was validated against groups of functional assay values, human disease cases, and population genetics data. We present the list of variants most likely seen as future determinants of RAG deficiency, meriting functional investigation.

## Methods

### Population Genetics and Data Sources

GnomAD (version r2.0.2) [21] was queried for the canonical transcripts of *RAG1* and *RAG2* from population genetics data of approximately 146,000 individuals; ENST00000299440 (*RAG1*) 1586 variants, GRCh37 11:36532259-36614706 and ENST00000311485 (*RAG2*) 831 variants, GRCh37 11:36597124 - 36619829. Data was filtered to contain the variant effect identifiers: frameshift, inframe deletion, inframe insertion, missense, stop lost, or stop gained. Reference transcripts were sourced from Ensembl in the FASTA format amino acid sequence for transcript RAG1-201 ENST00000299440.5 [HGNC:9831] and transcript RAG2-201 ENST00000311485.7 [HGNC:9832]. These sequences were converted to their three-letter code format using *One to Three* from the Sequence Manipulation Suite (SMS2) [24]. Combined Annotation Dependent Depletion (CADD) scores were sourced from https://cadd.gs.washington.edu/download (Nov 2018) and are reported by Kircher et al. [25]. The dataset used was “All possible SNVs” from whole-genome data, from which we extracted the data for coding regions of RAG1 and RAG2. We used the Human Gene Mutation Database (HGMD) from the Institute of Medical Genetics in Cardiff as a pre-defined source of known RAG deficiency cases http://www.hgmd.cf.ac.uk/ac/index.php [26] (Feb 2019, free access version to NM_000448.2). Data was formatted into CSV and imported into R for combined analysis with PHRED-scaled CADD scores and the main data frame. The crystal structure render of DNA-bound RAG complex was produced with data from RCSB Protein Data Bank (3jbw.pdb) [27]. Structures were visualized using the software VMD from the Theoretical and Computational Biophysics Group [28], imaged with Tachyon rendering [29], and color mapped using our scoring method.

### Data Processing

The population genetics input dataset used GnomAD variant allele frequencies and reference sequences processed as CSV files and cleaned and sorted to contain only amino acid codes, residue numbers, alternate residues, alternate allele frequencies, and a score of 0 or 1 to indicate presence or absence of variants where 1 represented none reported. An annotation column was also provided to label where multiple alternate variants existed. Statistics and calculation steps are listed in order in Supplemental Tables E3–E8.

The percentage of conserved residues was calculated (55.99% of amino acids contained no reported variants in RAG1, 55.98% in RAG2 (Table E4)). Basic protein statistics were generated using canonical reference transcript sequences of RAG1 and RAG2 with the SMS2 tool *Protein Stats* [24]. The resulting pattern percentage value was converted to a frequency (decimal 0–1) based on the number of residues per protein to generate the residue frequency (*Rf*). The *Rf* values were found for both proteins as shown in Table E5 and summarized in Table E6.

The count of variants per residue was found for both proteins, and the mutation rates (*Mr*) per residue were calculated as shown in Table E7. *Mr* was found by counting the number of mutations per residue in a window, sized to contain each protein individually. For genome-wide application, the window size may be increased or decreased. In this case, the window consisted of only the coding regions. The *Mr* values were then converted to frequencies based on the number of residues per protein. Separate, and overlapping, windows could also be used based on genome phase data and regions of linkage disequilibrium to account for non-random association of alleles at different loci; this might be particularly important for disorders with multiple genetic determinants.

**Fig. 1:**
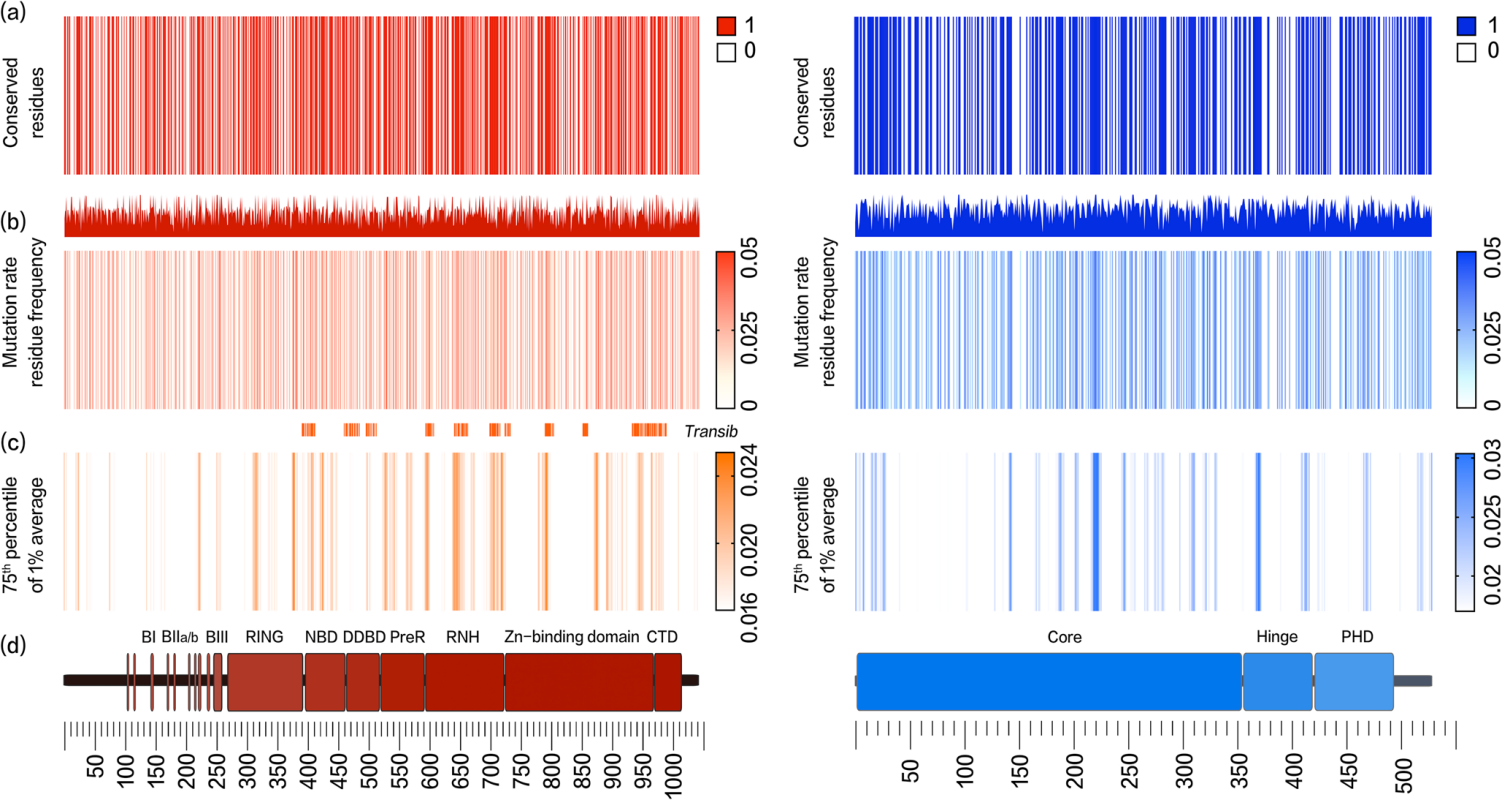
RAG1 (red, left) and RAG2 (blue, right) conservation and mutation rate residue frequency. **a** Gene conservation score, non-conserved 0 and conserved 1. The color indicates no known mutations in humans. **b** Histogram, raw MRF score; Heatmap, MRF prediction for conserved residues, graded 0 to 0.05 (scale of increasing mutation likelihood with human disease). **c** Colored bars indicate most likely clinically relevant variant clusters. MRF score averaged with 1% intervals for each gene and cutoff below the 75th percentile, graded 0 to 0.03 (noise reduction method). **d** Gene structure with functional domains. Full list of residues and scores available in Table E1.

The *Mr* and *Rf *multiply to give the raw mutation rate residue frequency (MRF) value (Table E8). This value is also shown in Tables 1 and E1. Our investigation used a Boolean score *C* to account for the presence or absence of a mutation in the general population, 0 for any variant existing in the population and 1 for conserved residues. *C* × *Mr* × *Rf*, in our case, produced the MRF score for conserved residues. Figure 1a illustrates the raw MRF as a histogram and the MRF, after applying C, as a heatmap.

**Table 1:** MRF likelihood scores for variants functionally assayed to date [16–18]. Increased MRF score indicates a higher likelihood of occurrence. Recombination activity is shown as a percentage of wild type (% SEM). Residues with multiple mutations are shown with both alternative variants and values. MRFmax = 0.043 and MRFmin = 0.004. The full table of all protein positions can be found in Supplemental Table E1

**RAG1**			
**MRF**	**Residue**	**Assayed**	**Recombination activity (%)**
0.03	56	I56T	3.5 ± 0.2
0.03	86	K86VfsX33	2.7 ± 0.3
0.014	99	G99S	113.2 ± 3.7
0.012	106	N106K	80.4 ± 16.4
0.043	108	R108X	1.8 ± 0.3
0.043	142	R142X	9.0 ± 4.0
0.032	174	E174SfsX27	0.5 ± 0.2
0.027	246	A246TfsX17	0.8 ± 0.1
0.012	248	Q248X	1.2 ± 0.2
0.026	249	H249R	112.2 ± 3.5
0.043	314	R314W	24.3 ± 5.2
0.012	328	C328Y	16.0 ± 2.9
0.03	383	K383RfsX7	0.1 ± 0.0
0.013	386	F386CfsX4<a0>	0.2 ± 0.1
0.03	391	K391E	6.5 ± 1.6
0.043	394	R394Q	0.1 ± 0–0.1
0.043	396	R396C	0.4–0.6 ± 0–0.1
0.041	401	S401P	0.0 ± 0.0
0.02	403	T403P	0.0 ± 0.0
0.043	404	R404Q	1.2 ± 0.1
0.043	410	R410Q	0.0 ± 0.0
0.025	411	L411P	0.0 ± 0.0
0.022	429	D429G	0.1 ± 0.0
0.028	433	V433 M	0.2 ± 0.0
0.019	435	M435 V	23.6 ± 4.8
0.027	444	A444V	1.4 ± 0.2
0.043	449	R449K	92.1 ± 3.6
0.025	454	L454Q	5.4 ± 0.7
0.019	458	M458SfsX34	0.0 ± 0.0
0.027	472	A472V	0.4 ± 0.0
0.043	474	R474C	125.4 ± 2.6
0.028	475	V475AfsX17	0.1 ± 0.0
0.025	506	L506F	1.0 ± 0.1
0.043	507	R507W	15.9 ± 0.8
0.014	516	G516A	40.2 ± 1.3
0.005	522	W522C	41.6 ± 1.9
0.022	539	D539V	3.2 ± 0.2
0.025	541	L541CfsX30	1.2 ± 0.9
0.043	559	R559S	1.0 ± 0.4
0.043	561	R561H	2.0 ± 0.6
0.041	601	S601P	0.0 ± 0.0
0.026	612	H612R	121.6 ± 0.9
0.043	624	R624H	0.0 ± 0.4
0.041	626	S626X	0.0 ± 0.0
0.041	651	S651P	0.5 ± 0.5
0.043	699	R699Q,W	45.9 ± 1.5, 19.3 ± 1.8
0.032	722	E722K	0.0 ± 0.2
0.012	730	C730F	0.0 ± 0.0
0.025	732	L732P	0.0 ± 0.0
0.043	737	R737H	0.2 ± 0.0
0.043	759	R759C	17.2 ± 3.3
0.043	764	R764P	0.0 ± 0.0
0.008	768	Y768X	0.0 ± 0.0
0.032	770	E770K	21.0 ± 0.4
0.043	778	R778Q,W	8.6 ± 1.0, 4.6 ± 0.6
0.028	786	P786L	0.0 ± 0.1
0.03	820	K820R	117.9 ± 6.3
0.025	836	L836 V	75.0 ± 1.3
0.043	841	R841Q,W	0.0 ± 0.0, 10.0 ± 0.5
0.027	868	A868V	100.0 ± 5.0
0.005	896	W896R	0.9 ± 0.1
0.008	912	Y912C	6.9 ± 0.4
0.005	959	W959X	0.0 ± 0.0
0.032	965	E965X	0.0 ± 0.0
0.043	973	R973C	0.0 ± 0.2
0.013	974	F974 L	56.5 ± 0.8
0.043	975	R975W,Q	57.9 ± 1.6, 53.5 ± 3.6
0.012	981	Q981P	7.2 ± 0.1
0.03	983	K983NfsX9	0.1 ± 0.0
0.03	992	K992E	9.1 ± 1.2
0.03	1006	M1006 V	105.6 ± 6.8
**RAG2**			
0.013	1	M1T	65.3 ± 2.2
0.006	16	Q16X	1.7 ± 0.4
0.038	35	G35A,V	22.1 ± 3.1, 0.4 ± 0.3
0.023	39	R39G	0.2 ± 0.1
0.011	41	C41W	0.2 ± 0.4
0.017	62	F62 L	19.6 ± 3
0.028	65	D65Y	6.8 ± 1.2
0.023	73	R73H	12.4 ± 1.4
0.034	77	T77 N	42.6 ± 2.7
0.038	95	G95R	0.3 ± 0.2
0.013	110	M110 L	74.6 ± 1.8
0.017	127	K127X	0.1 ± 0
0.038	157	G157 V	0.4 ± 0.2
0.03	160	S160 L	5.8 ± 0.6
0.023	180	P180H	31.1 ± 0.5
0.019	195	Y195D	2 ± 0.3
0.034	215	T215I	67.2 ± 1
0.023	229	R229Q,W	8.9 ± 1, 10.5 ± 0.5
0.023	253	P253R	95.4 ± 2.3
0.006	278	Q278X	0.1 ± 0.1
0.013	285	M285R	24.7 ± 0.8
0.004	307	W307X	0.2 ± 0.2
0.017	386	F386 L	109.1 ± 5
0.025	407	E407X	2.9 ± 0.4
0.004	416	W416 L	1.4 ± 0.2
0.025	437	E437K	0.9 ± 0.2
0.017	440	K440 N	26.7 ± 2.4
0.013	443	M443I	0.4 ± 0.2
0.027	444	I444M	2.7 ± 0.3
0.011	446	C446W	2.9 ± 0.1
0.038	451	G451A	66.3 ± 4.8
0.004	453	W453R	0.6 ± 0.1
0.017	456	A456T	16 ± 2.9
0.013	459	M459 L	30.8 ± 0.6
0.034	474	N474S	97.5 ± 5.9
0.011	478	C478Y	0.2 ± 0.1
0.025	480	E480X	2.8 ± 0.6
0.017	481	H481P	23.8 ± 3.9
0.013	502	M502 V	99.6 ± 3.4

An important consideration for future application is whether to use this Boolean score or instead use a discrete variable which accounts for the true allele frequency in the general population. In the clinical setting, the likelihood of de novo mutations and inherited mutations have different impacts when considering recessive and dominant diseases. A patient is more likely to inherit a variant that exists even at a very low frequency than to acquire a random de novo mutation. Therefore, a value representing an allele frequency may be used to replace *C* in many investigations, particularly when considering variants that exist at low rates. PRHED-scaled CADD score data consisted of nucleotide level values. For comparison with MRF, the median CADD scores were averaged per codon as demonstrated in Supplemental text. A summary of data processing and analysis is illustrated in Fig. E1.

### Raw Data Availability and Analysis Script

The Supplemental “Raw_data_R_analysis_for_figures” contains all raw data and analysis methods used to produce figures (except illustrations in Figs. 1 and 6). “data_analysis.R” is an R script that contains the methods used to produce figures. Each of the input data CSV files is explained on the first usage within the analysis script. Running “data_analysis.R” from within the same directory as the associated input data CSV files will replicate analysis.

### Data Visualization

For our visualization of MRF scores, small clusters of high MRF values were more appealing than individual highly conserved residues. Therefore, we applied a 1% average filter where values were averaged over a sliding window of N number of residues (10 in the case of RAG1, 6 in the case of RAG2). For a clear distinction of MRF clusters, a cutoff threshold was applied at the 75th percentile (e.g., 0.0168 in RAG1) as shown in heatmaps in Figs. 1c and 6. The gene heatmaps for coding regions in RAG1 and RAG2 (Fig. 1) were populated with (i) Boolean *C* score from population genetics data, (ii) raw MRF scores, and (iii) MRF clusters with 1% average and cutoff threshold. GraphPad Prism was used for heatmaps. The data used for heatmaps is available in Table E1 and in the supplemental R source to allow for alternative visualizations. An example of alternative output for non-R users is shown in Fig. E2. Adobe Illustrator and Photoshop were used for protein domain illustrations in Fig. 1d. Data and analysis is summarized in Fig. E1.

### Validation of MRF Against Functional Data

The recombination activity of RAG1 and RAG2 was previously measured on known or candidate pathogenic variants [16–18]. Briefly, the pathogenicity of variants in RAG1 and RAG2 was measured functionally in vitro by either expression of RAG1 and RAG2 in combination with a recombination substrate plasmid containing recombination signal sequence (RSS) sites which are targeted by RAG complex during normal V(D)J recombination, or Abelson virus-transformed Rag2−/− pro-B cells with an RSS-flanked inverted GFP cassette. Recombination events were assessed by quantitative real-time PCR using comparative CT or expression of GFP evaluated by flow cytometry, respectively. The inverse score of recombination activity (0–100%) was used to quantify pathogenicity of variants in our study. Comparison between known pathogenicity scores and MRF was done by scaling MRF scores from 0 to 100% (100% being the highest probability of occurring as damaging).

## Results

### RAG1 and RAG2 Conservation and Mutation Rate Residue Frequency

Variant probability prediction is dependent on population genetics data. Our study queried GnomAD [21] to identify conserved residues using a Boolean score *C* of 0 (present in population) or 1 (conserved). The gene-specific mutation rate *Mr* of each residue was calculated from variant allele frequencies. The gene-specific residue frequency *Rf* represented the frequency of a residue occurring per gene, acquired by converting gene residue percentage (from the SMS2 tool *Protein Stats*) to a frequency (decimal 0–1) [24]. Together, the values were used to calculate the most probable disease-causing variants which have not yet been identified in patients. We termed the resulting score a mutation rate residue frequency, where *MRF* = *C* × *Mr* × *Rf*. This score represents the likelihood that a clinically relevant mutation will occur.

Figure 1 presents the most probable unidentified disease-causing variants in RAG1/2. Variants with a low MRF may still be damaging, but resources for functional validation are best spent on gene regions with high MRF. Clusters of conserved residues are shown in Fig. 1a and are generally considered important for protein structure or function. However, these clusters do not predict the likelihood of mutation. Raw MRF scores are presented in Fig. 1b. Histograms illustrate the MRF without Boolean scoring applied and Fig. 1c provides a clearer illustration of top MRF score clusters. For visualization, a noise reduction method was applied; a sliding window was used to find the average MRF per 1% interval of each gene. The resulting scores displayed in Fig. 1c contain a cutoff threshold to highlight the top-scoring residues (using the 75th percentile). Variant sites most likely to present in disease cases are identified by high MRF scoring. This model may be expanded by the addition of phenotypic or epigenetic data (Supplemental; *Bayesian probability*).

Table E1 provides all MRF scores for both proteins. Raw data used for calculations and the list of validated residues of RAG1 and RAG2 are available in Tables E3–E8. Table 1 shows the MRF mutation likelihood score for mutations that have also been reported as tested for recombination activity in functional assays. The likelihood of mutation does not correlate with pathogenicity; Figs. 3 and E3 show that most mutations tested had severe loss of protein function, while the likelihood of each mutation occurring in humans varied significantly. Analysis-ready files are also available in Supplemental data along with the associated R source file to allow for alternative visualizations as shown in Fig. E2.

### MRF Scores Select for Confirmed Variants in Human Disease

**Fig. 2:**
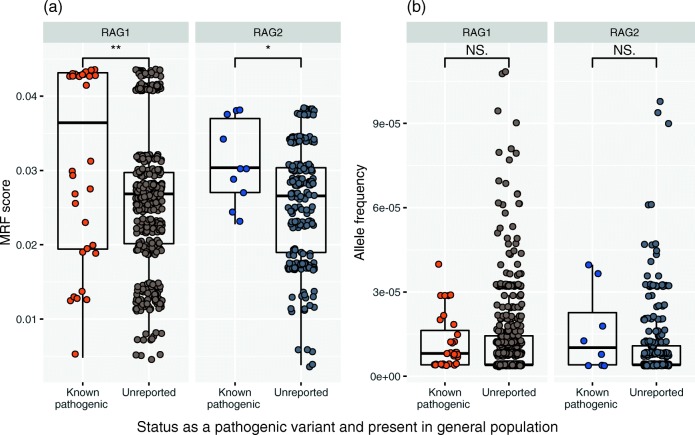
RAG1 and RAG2 MRF scores predict the likelihood of mutations that are clinically relevant. **a** Known damaging variants (clinically diagnosed with genetic confirmation) reported on GnomAD have significantly higher MRF scores than unreported variants. **b** GnomAD rare variant allele frequency < 0.0001. No significant difference in allele frequency is found between known damaging and non-clinically reported variants. Unpaired *t* test, RAG1 *P* value 0.002** and RAG2 *P* value 0.0339*. MRF, mutation rate residue frequency; ns, non-significant

We have applied MRF scores to known damaging mutations from other extensive reports in cases of human disease [12, 15, 17, 19, 20, 30–53] (originally compiled by Notarangelo et al. [54]). This dataset compares a total of 44 variants. We expected that functionally damaging variants (resulting in low recombination activity in vitro) that have the highest probability of occurrence would be identified with high MRF scores. MRF prediction correctly identified clinically relevant mutations in *RAG1* and *RAG2* (Fig. 2a). Variants reported on GnomAD which are clinically found to cause disease had significantly higher MRF scores than variants which have not been reported to cause disease. We observed that rare and likely mutations provided high scores while rare but unlikely or common variants had low scores (Fig. 2b).

Allele frequency is generally the single most important filtering method for rare disease in whole-genome (and exome) sequencing experiments. Variants under pressure from purifying selection are more likely to cause disease than common variants. However, most RAG mutations are rare. Therefore, allele frequencies of rare variants reported on GnomAD cannot differentially predict the likelihood of causing disease (Fig. 2b). As such, we found no significant difference between known damaging variants and those that have not yet been reported as disease causing. The comparison between Fig. 2a and b illustrates the reasoning for the design of our method.

Many non-clinically reported rare variants may cause disease; the MRF score identifies the top clinically relevant candidates. Based on the frequency of protein-truncating variants in the general population, *RAG1* and *RAG2* are considered to be tolerant to the loss of one allele, as indicated by their low probability of being loss-of-function intolerant (pLI) scores of 0.00 and 0.01, respectively [21]. This is particularly important for recessive diseases such as RAG deficiency where most new missense variants will be of unknown significance until functionally validated.

### Top Candidate Variants Require Validation

Functionally, characterizing protein activity is both costly and time consuming. RAG1 and RAG2 have now been investigated by multiple functional assays for at least 110 coding variants [16–18]. In each case, researchers selected variants in *RAG1* and *RAG2* that were potentially damaging or were identified from PID patients as the most probable genetic determinant of disease. Functional assays for RAG deficiency in those cases, and generally, measured a loss of recombination activity as a percentage of wild-type function (0–100%).

**Fig. 3:**
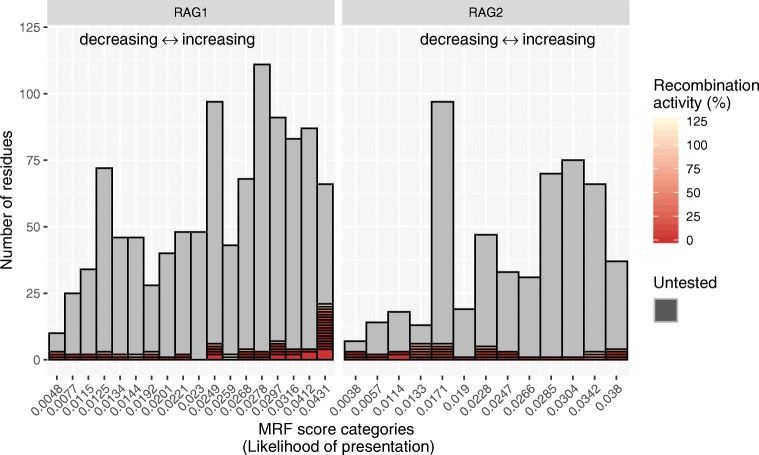
RAG1 and RAG2 MRF score categories and variants assayed to date. Protein residues are ranked and stacked into categories based on their MRF score. High scores (0.043 and 0.038 in RAG1 and RAG2, respectively) represent a greater mutation likelihood. Functional assays have measured recombination activity (as its inverse; % loss of activity) in a total of 110 mutants. The severity of protein loss of function is represented by a red gradient. Residues that have not been functionally tested are shown in grey. While many protein residues are critical to protein function, their mutation is less probable than many of the top MRF candidates. Data further expanded in Fig. E3. MRF, mutation rate residue frequency

Pre-emptively, performing functional variant studies benefits those who will be identified with the same variants in the future, before the onset of disease complications. While more than 100 variants have been assayed in vitro, we calculated that only one-quarter of them are most probable candidates for clinical presentation. Figure 3 illustrates that while functional work targeted “handpicked” variants that were ultimately confirmed as damaging, many of them may be unlikely to arise based on population genetics data. Figure 3 presents, in increasing order, the number of potential variants based on the likelihood of presentation and stacked by the number of variants per score category. Variants that have been measured for their loss of protein activity are colored by severity. Potential variants that remain untested are colored in grey. Only 21 of the top 66 most probable clinically relevant variants have been assayed in RAG1.

Supplemental Fig. E3 further illustrates the individual variants which have been tested functionally (the colored recombination activity subset of Fig. 3). We compared predicted MRF scores to assay measurements for 71 RAG1 and 39 RAG2 mutants. Most mutations tested showed severe loss of protein function (bottom panel of Supplemental Fig. E3), while the likelihood of each mutation occurring in humans varied significantly (top panels).

If MRF scoring was used in the same cases pre-emptively, the loss of investment would be minimal; only 8 variants out of 71 mutants tested had an above-average MRF score while being measured as functionally benign (a rate of 11.27%). RAG2 had only 3 out of 39 variants (7.69%) with an above-average MRF score while functionally benign. For the expended resources, approximately 30% more top candidates would have been tested in place of unlikely and functionally non-damaging mutations. However, the true measurement of accuracy is limited in that very few of the most likely clinically relevant variants predicted by MRF scoring have been tested to date.

### False Positives in Transib Domains Do Not Negatively Impact Prediction

Adaptive immunity is considered to have evolved through jawed vertebrates after integration of the RAG transposon into an ancestral antigen receptor gene [55, 56]. The Transib transposon is a 600 amino acid core region of RAG1 that targets RSS-like sequences in many invertebrates. A linked RAG1/RAG2 was shown in the lower dueterostome (sea urchin), indicating an earlier common ancestor than the invertebrate [57], and more recently, a recombinatorially active RAG transposon (ProtoRAG) was found in the lower chordate amphioxus (or lancelet), the most basal extant chordate and a “living fossil of RAG” [58].

**Fig. 4:**
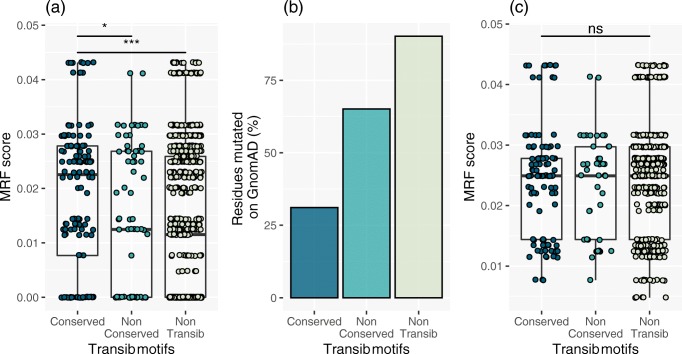
False positives in Transib domains do not worsen probability prediction. The Transib domains contain critical conserved protein residues. **a** False positives were simulated by scoring Transib domains MRF without omitting Boolean conservation weight *C* = 0. **b** Allele frequencies on GnomAD had conservation levels inversely proportional to simulated false-positive MRF scoring. **c** When testing for all Boolean component *C* > 0 after MRF calculation, the effect of false positives remained non-significant, illustrating the non-negative impact of MRF for predicting the mutation rate. Unpaired *t* test, *P = 0.0195 and ***P < 0.0001. MRF, mutation rate residue frequency; ns, non-significant

A set of conserved motifs in core RAG1 are shared with the Transib transposase, including the critical DDE residue catalytic triad (residues 603, 711, and 965) [59]. Ten RAG1 core motifs are conserved amongst a set of diverse species including human [59]. This evolutionarily conserved region is considered as most important to protein function. Therefore, we chose this region to determine if MRF scoring would have a negative impact if mutations were falsely predicted as clinically important. To assess the influence of a false-positive effect on prediction, the MRF scores for conserved residues in this group were compared with GnomAD allele frequencies. Figure 4a plots the MRF (without omitting the Boolean component *C* = 0) for conserved Transib motif residues, non-conserved Transib motif residues, and non-Transib residues. Figure 4b shows the percentage of these which were reported as mutated on GnomAD. By accounting for unreported variants by applying *C* > 0, the resulting effect on incorrectly scoring MRF in the conserved Transib motifs remained neutral.

### MRF Predicts RAG Deficiency Amongst PID Patients Harboring Rare Variants

We have previously measured the recombination activity of RAG1 and RAG2 disease-causing variants in several patients [16]. We have compiled our own and other functional assay data from Lee et al. [17] and Tirosh et al. [18] to produce a panel of recombination activity measurements for coding variants in both RAG1 and RAG2. RAG deficiency was measured as the level of recombination potential produced by the protein complex. Each method of investigation simulated the efficiency of wild-type or mutant proteins expressed by patients for their ability to produce a diverse repertoire of T cell receptor (TCR) and B cell receptor (BCR) and coding for immunoglobulins. In functional experiments, mutant proteins were assayed for their ability to perform recombination on a substrate which mimics the RSS of TCR and BCR in comparison with wild-type protein complex (as % SEM).

**Fig. 5:**
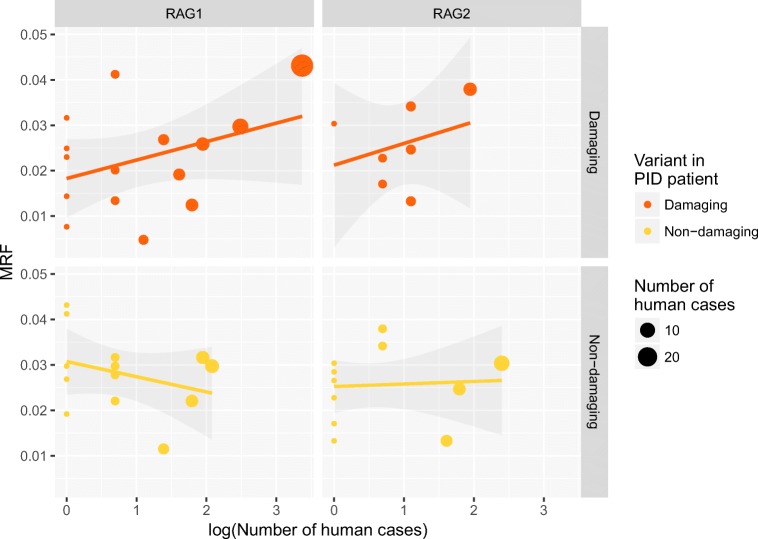
A linear regression model of RAG1/2 MRF scoring in cases of primary immune deficiency. MRF prediction correlates with clinical presentation. Damaging variants identified in confirmed RAG deficiency cases. Non-damaging variants sourced from cases of PID with rare variants but not responsible for disease. An MRF > 0.04 was seen for 31 cases of damaging RAG1 variants. (Slopes of RAG1: Damaging, 0.0008* (± 0.0004) *P* < 0.05, intercept 5.82e-05***; non-damaging, − 0.0007 (± 0.001). Slopes of RAG2: Damaging, 0.0023 (± 0.0018), intercept 0.0312*; non-damaging 0.0001 (± 0.0008). Source data and script in Supplemental material)

By gathering confirmed RAG deficiency cases, we compiled the MRF scores for 43 damaging RAG1 variants in 77 PID cases and 14 damaging RAG2 variants in 21 PID cases (MRF scores spanning over 22 categories). To test our method against a strong control group, we identified coding variants in patients with PID where RAG deficiency due to coding variants has been ruled out as the cause of disease. We obtained RAG1/2 variants in 558 PID patients who had their genomes sequenced as part of the NIHR BioResource - Rare Diseases study [16]. Filtering initially identified 32 variants in 166 people. This set was trimmed to contain only rare variants; 29 variants over 26 MRF scoring categories from 72 cases of non-RAG-deficient PID. The scatterplot in Fig. 5 shows that most PID cases had damaging variants with a high MRF score, while PID cases carried benign variants in RAG1/2 with lower MRF scores, i.e., an MRF > 0.04 was seen for 31 cases of a damaging variant and only 2 cases of a non-damaging variant. Linear regression on this control group produced negative or near-zero slopes for RAG1 and RAG2, respectively. The same analysis for known damaging mutations in disease cases had significant prediction accuracy for RAG1. Analysis of RAG2 was not significant. However, the sample size to date may be too small to significantly measure RAG2 MRF scoring although a positive correlation was inferred in Fig. 5 [60]. R source and raw data can be found in Supplemental material.

### MRF Supplements Pathogenicity Prediction Tools for Translational Research

**Fig. 6:**
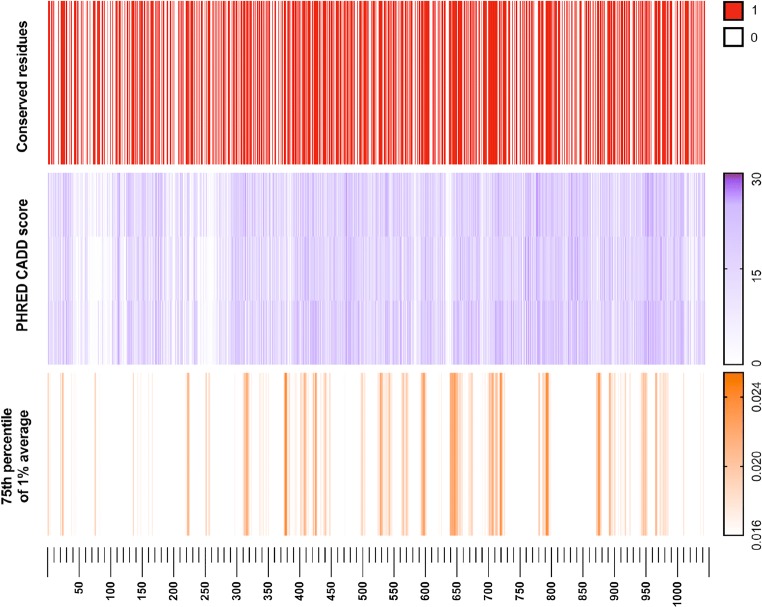
RAG1 PHRED-scaled CADD score versus GnomAD conservation rate and MRF score. Allele frequency conservation rate (top) is vastly important for identifying critical structural and functional protein regions. The impact of mutation in one of these conserved regions is often estimated using CADD scoring (middle). CADD score heatmap is aligned by codon and separated into three layers for individual nucleotide positions. The MRF score (bottom) (visualized using the 75th percentile with 1% averaging) highlights protein regions that are most likely to present clinically and may require pre-emptive functional investigation

CADD scoring [25] is an important bioinformatics tool that exemplifies pathogenicity prediction. While CADD is a valuable scoring method, its purpose is not to predict the likelihood of variation. Similarly, MRF scoring is not a measure of pathogenicity. MRF scoring may be complemented by tools for scoring variant deleteriousness. We compare MRF to the PHRED-scaled CADD scores for all possible SNV positions in *RAG1* (Fig. 6) illustrating that pathogenicity prediction cannot account for mutation probability. Combining both methods allows researchers to identify highly probable mutations before querying predicted pathogenicity.

**Fig. 7:**
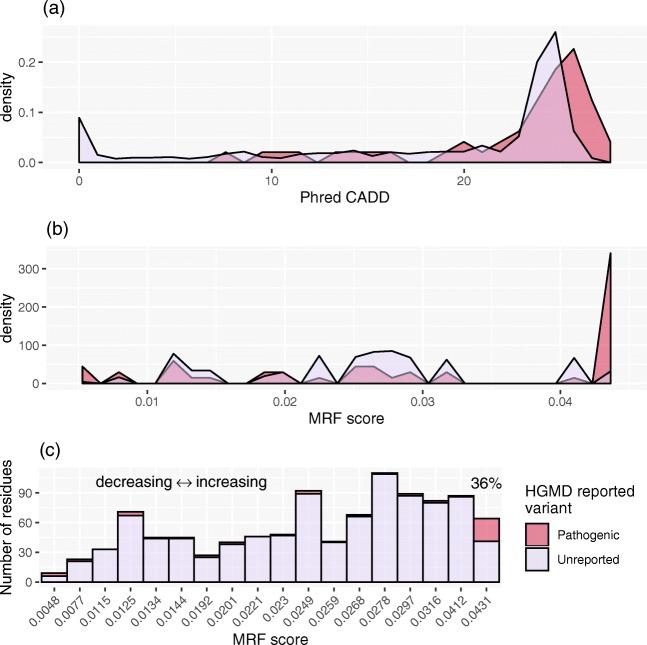
RAG1 PHRED-scaled CADD score versus MRF score against HGMD data. **a** A high CADD score is a predictor of deleteriousness. Both reported (red) and non-reported residues (pink) have a high density of high CADD score. **b** MRF scores only show a high-density cluster for high-likelihood variants, reflected by the high MRF score observed for known RAG deficiency variants. The number of pathogenic variants is outweighed by conserved residues; **a**, **b** shows the density of scores to normalize between groups. AUC overlap difference in CADD score of 21.43% and MRF score of 74.28% (above intersects > 22.84 and > 0.0409, in **a** and **b** respectively). **c** The number of residues per MRF category shows that disease reported on HGMD accounts for 36% of top MRF candidates. AUC, area under curve; CADD, Combined Annotation Dependent Depletion; HGMD, Human Gene Mutation Database

To further develop this concept, we firstly annotated variants with MRF likelihood scores and pathogenic prediction PHRED-scaled CADD scores (Fig. 7) and secondly performed a manual investigation of the clinical relevance of top candidates (Table E2). We used HGMD as an unbiased source of known RAG deficiency cases in both instances. CADD score was very successful at predicting the pathogenicity of a variant, (a high-density cluster of variants with CADD scores > 25) as shown in red in Fig. 7a. At about the same rate, CADD score also predicted variants as pathogenic that are, to date, unreported (as pink in Fig. 7a). Indeed, those unreported variants may very well be pathogenic. However, the likelihood of each mutation varies. As such, we developed the MRF score to account for that likelihood. As expected, the likelihood of mutations occurring that were unreported was low according to MRF (Fig. 7b, pink), while the mutations which did occur were highly enriched in at high MRF scores (Fig. 7b, red high-density cluster > 0.043). Combining mutation prediction (MRF) with pathogenicity prediction (tools like CADD) increases the accuracy of pre-emptively targeting clinically relevant variants. Figure 7c shows that while the number of variants presented to date is relatively small, they already account for 36% of the top MRF score candidates.

## Discussion

Determining disease-causing variants for functional analysis typically aims to target conserved gene regions. On GnomAD, 56% of *RAG1 *(approx. 246,000 alleles) is conserved with no reported variants. Functional validation of unknown variants in genes with this level purifying selection is generally infeasible. Furthermore, we saw that a vast number of candidates are “predicted pathogenic” by commonly used pathogenicity tools, which may indeed be damaging but unlikely to occur. To overcome the challenge of manual selection, we quantified the likelihood of mutation for each candidate variant.

Targeting clearly defined regions with high MRF scores allows for functional validation studies tailored to the most clinically relevant protein regions. An example of high MRF score clustering occurred in the RAG1 catalytic RNase H (RNH) domain at p.Ser638-Leu658 which is also considered a conserved Transib motif.

While many hypothetical variants with low MRF scores may be uncovered as functionally damaging, our findings suggest that human genomic studies will benefit by first targeting variants with the highest probability of occurrence (gene regions with high MRF). Table E1 lists the values for calculated MRFs for RAG1 and RAG2.

We have presented a basic application of MRF scoring for RAG deficiency. The method can be applied to genome wide. This can include phenotypically derived weights to target candidate genes or tissue-specific epigenetic features. In the state presented here, MRF scores are used for pre-clinical studies. A more advanced development may allow for use in single cases. During clinical investigations using personalized analysis of patient data, further scoring methods may be applied based on disease features. A patient phenotype can contribute a weight based on known genotype correlations separating primary immunodeficiencies or autoinflammatory diseases [6]. For example, a patient with autoinflammatory features may require a selection that favors genes associated with proinflammatory diseases such as *MEFV*and *TNFAIP3*, whereas a patient with mainly immunodeficiency may have preferential scoring for genes such as *BTK* and* DOCK8*. In this way, a check-list of most likely candidates can be confirmed or excluded by whole genome or panel sequencing. However, validation of these expanded implementations requires a deeper consolidation of functional studies than is currently available.

Havrilla et al. [61] have recently developed a method with similar possible applications for human health mapping constrained coding regions. Their study employed a method that included weighting by sequencing depth. Similarly, genome-wide scoring may benefit from mutation significance cutoff, which is applied for tools such as CADD, PolyPhen-2, and SIFT [62]. We have not included an adjustment method as our analysis was gene-specific but implementation is advised when calculating genome-wide MRF scores.

The MRF score was developed to identify the topmost probable variants that have the potential to cause disease. It is not a predictor of pathogenicity. However, MRF may contribute to disease prediction; a clinician may ask for the likelihood of RAG deficiency (or any other Mendelian disease of interest) prior to examination (Supplemental)[68].

Predicting the likelihood of discovering novel mutations has implications in genome-wide association studies (GWAS). Variants with low minor allele frequencies have a low discovery rate and low probability of disease association [63], an important consideration for rare diseases such as RAG deficiency. An analysis of the NHGRI-EBI catalogue data highlighted diseases whose average risk allele frequency was low [63]. Autoimmune diseases had risk allele frequencies considered low at approximately 0.4. Without a method to rank most probable novel disease-causing variants, it is unlikely that GWAS will identify very rare disease alleles (with frequencies < 0.001). It is conceivable that a number of rare immune diseases are attributable to polygenic rare variants. However, evidence for low-frequency polygenic compounding mutations will not be available until large, accessible genetics databases are available, exemplified by the NIHR BioResource Rare Diseases study [16]. An Interesting consideration when predicting probabilities of variant frequency is that of protective mutations. Disease risk variants are quelled at low frequency by negative selection, while protective variants may drift at higher allele frequencies [64].

The cost-effectiveness of genomic diagnostic tests is already outperforming traditional, targeted sequencing [1]. Even with substantial increases in data sharing capabilities and adoption of clinical genomics, rare diseases due to variants of unknown significance and low allele frequencies will remain non-actionable until reliable predictive genomics practices are developed. Bioinformatics as a whole has made staggering advances in the field of genetics [65]. Challenges that remain unsolved, hindering the benefit of national or global genomics databases, include DNA data storage and random access retrieval [66], data privacy management [67], and predictive genomics analysis methods. Variant filtration in rare disease is based on reference allele frequency, yet the result is not clinically actionable in many cases. Development of predictive genomics tools may provide a critical role for single-patient studies and timely diagnosis [23].

## Conclusion

We provide a list of amino acid residues for RAG1 and RAG2 that have not been reported to date but are most likely to present clinically as RAG deficiency. This method may be applied to other diseases with hopes of improving preparedness for clinical diagnosis.

## Electronic supplementary material

ESM 1 (DOCX 36 kb)

ESM 2 (PDF 259 kb)

ESM 3 (PDF 18 kb)

ESM 4 (PDF 8 kb)

ESM 5 (PNG 1642 kb)

ESM 6 (CSV 88 kb)

ESM 7 (CSV 458 bytes)

ESM 8 (CSV 1020 bytes)

ESM 9 (CSV 245 bytes)

ESM 10 (CSV 1 kb)

ESM 11 (CSV 1 kb)

ESM 12 (CSV 1 kb)

ESM 13 (CSV 1 kb)

ESM 14 (TXT 24.9 kb)

ESM 15 (CSV 29 kb)

ESM 16 (CSV 99 kb)

ESM 17 (CSV 23 kb)

ESM 18 (CSV 5 kb)

ESM 19 (CSV 1 kb)

ESM 20 (CSV 20 kb)

ESM 21 (CSV 1 kb)

ESM 22 (TXT 864 bytes)

ESM 23 (CSV 82 bytes)

ESM 24 (CSV 19 kb)

## Abbreviations

BCR: B cell receptor
CADD: Combined annotation dependent depletion
CID-G/A: Combined immunodeficiency with granuloma and/or autoimmunity
GWAS: Genome-wide association studies
HGMD: Human gene mutation database
Mr.: Mutation rate
MRF: Mutation rate residue frequency
PID: Primary immunodeficiency
pLI: Probability of being loss-of-function intolerant
RAG1: Recombination activating gene 1
Rf: Residue frequency
RNH: RNase H
RSS: Recombination signal sequence
SCID: Severe combined immunodeficiency
TCR: T cell receptor
