# Supplementary material for: Predicting the Occurrence of Variants in RAG1 and RAG2
Source: J Clin Immunol. 2019 Aug 6;39(7):688–701. doi: 10.1007/s10875-019-00670-z (PMC6754361; doi:10.1007/s10875-019-00670-z)
Supplement: Supplementary file 1 — (DOCX 36 kb) [file 10875_2019_670_MOESM1_ESM.docx]

Supplemental

Figure E1: Data analysis summary map. Raw data and analysis scripts are provided in the supplemental. Analysis steps and data sources for each procedure described in methods. MRF; mutation rate residue frequency, PID; primary immunodeficiency.

Figure E2: An alternative visualisation of MRF scores for RAG1 and RAG2 proteins. The data from Table E1 in column “Average over 1%” is displayed on both the y-axis and colour scale. An analysis-friendly long form CSV of the Table E1 data is also provided in the compressed supplemental R data “mrf.csv”.

Figure E3: MRF likelihood score versus known functional activity. We compiled all variants that we know to have been assayed for protein function to date. The inverse of functional assay measurements were used, where 0% activity represents 100% loss of activity. MRF scores are presented as a percentage of the maximum score per gene (i.e., for RAG1 M RFmax = 0.043 (100%) and M RFmin = 0.0048 (0%)). Top panels show how likely each mutation is predicted to occur in humans. Bottom panels show the loss of protein activity as a percentage compared to wild-type (% SEM); most mutations tested produced severe loss of protein function, regardless of their mutation likelihood. Subset of Recombination activity data from Figure 3.

Table E1: MRF data tables. The complete scores are listed for each protein. Both the wild type and alternative variants reported on GnomAD are shown [21]. Multiple alternative variants are reported for some residues which are annotated in a separate column. A column listing the 1% average of MRF scores using a sliding window is present; this is used in Figure 1 (iii) with a cut-off threshold at the 75th percentile to clearly visualise high scoring clusters. The Boolean conservation score is based on population genetics data, where 1 represents no known variant at a residue site. The percentile values for 0.9 and 0.75 are provided to allow for alternative visualisation methods using the R source supplemental “mrf.csv” file.

Clinical relevance of top candidates

The top scoring candidates in RAG1 were assessed for potential clinical relevance (Table E2). HGMD was chosen as a reliable, curated source of identifying pathogenic variants. 45% of RAG1 variants reported on HGMD (23 of 51) were predicted by our model as the most likely candidates seen clinically (the top scoring MRF group of had 66 residues total). The remaining variants in the top MRF group, which were not reported by HGMD (43 of 66), were assessed manually for their likelihood as potentially disease causing. 21 (49%) were highly conserved, not reported on GnomAD, and would be considered probable RAG deficiency on presentation as homozygous or compound heterozygous with a second damaging variant. The remainder had allele frequencies <0.0006, were only found as low frequency heterozygous in the general population, and justify functional validation. We expect that none of the top candidate mutations are benign.

Table E2: Clinical relevance of top candidates. 23 top MRF score variants were reported as pathogenic on HGMD to date. The remaining variants (the 43 not reported) were assessed by their frequency in population based on GnomAD (allele frequencies vary between individual variants but equate to approximately <6* and 9-77** heterozygous per 125,000 individuals). Therefore, no top candidates should be considered benign without functional validation. HGMD; Human Gene Mutation Database, MRF; mutation rate residue frequency.

Supplemental data tables

Table E3: Known damaging in human cases. Amino acids and residue numbers are listed along with their basic mutation rate residue frequency value for known RAG deficiency [54]. Supplemental CSV file.

Table E4: Percentage of variants per gene. Percentage of mutated versus non-mutated amino acids in RAG1 and RAG2 based on GnomAD population genetics data [21]. Supplemental CSV file.

Table E5: Residue frequency. Basic statistics for RAG1/2 were produced using SMS2 [24]. Results are shown for the canonical sequences, a 1043-residue sequence of RAG1-201 peptide ENSP00000299440 and for a 527-residue sequence of RAG2-201 peptide ENSP00000308620. Both percentage and frequency of residue usage are provided. Supplemental CSV file.

Table E6: Simplified residue frequency. The data from Table E5 is simplified for use in data analysis to only include residues, count, total, and frequency per protein expressed. Supplemental CSV file.

Table E7: Ratio of mutation per gene. The number of times each residue is mutated was found in population genetic data. The number of mutant versus wild-type is shown, from which the rate of each is derived. Supplemental CSV file.

Table E8: Basic MRF scores. The mutation rate and frequency is shown which were used to calculate the basic mutation rate residue frequency for the main analysis dataframe and Table E1. Supplemental CSV file.

Protein structure application

With the availability of a structured protein complex, modelling can be carried out prior to functional assays. Residues with the highest MRF for both RAG1 and RAG2 were mapped in Figure E4.

Figure E4: The RAG1 (blue) and RAG2 (grey) protein structure with top candidate MRF scores. (i) Protein dimers and (ii=iv) monomers illustrating the three highest category MRF scores for predicted clinically relevant variants. Increasing in score the top three MRF categories (illustrated in Figure 3) for each protein are highlighted; yellow, orange, red. DNA (green) is bound by the RAG protein complex at recombination signal sequences. DNA contact points are integral to protein function. (PDB:3jbw)

Median CADD score per residue

The sourced PHRED-scaled CADD score data consisted of nucleotide level values. We were interested in CADD scores averaged per codon. For every nucleotide position there were three alternative variants to consider, e.g.

Chrom Pos Ref Alt1 Alt2 Alt3 PHRED1 PHRED2 PHRED3

11 36594855 A C G T 22.3 18.81 22.4

The PHRED-scaled scores are listed here; raw CADD scores are also included in the original database. To produce a working input we used the median score per codon, that is three scores per nucleotide and three nucleotides per codon. This produced median PHRED-scaled score per codon / residue, e.g.:

Chrom Pos PHRED1 PHRED2 PHRED3

11 36594855 22.3 18.81 22.4

11 36594856 25.3 24.8 23.6

11 36594857 24.6 24.3 24.5

Median PHRED = 24.3

Supplemental file ‘RAG1.cadd.amino.csv’ within the analysis data ‘Raw_data_R_analysis_for_figures’ contains the median values over a three-nucleotide window, starting at nucleotide 1 to produce input data with the correct reading frame. The “PHRED-scaled” values are used as a normalised and externally comparable unit of analysis, rather than raw CADD scores. The area under the curve was calculated for density plots to quantify the difference between pathogenic and unreported variants with high scores, above the intersects >0.0409 and >22.84 for MRF and CADD, respectively, using score value (x) versus density (y) (Fig.7(i-ii)) with ∫_a^b▒〖□(24&f(x)dx≈(b-a)) [(f(a)+f(b))/2] 〗 (FOMRULA formatted in LaTeX version).

Genome-wide and disease-specific application

Weighting data can also be applied to the MRF score model to amplify the selectivity. The mutation rate can be applied genome wide with a process common in the study of information retrieval; term frequency, inverse document frequency (tf-idf). In this case the “term” and “document” are replaced by amino acid residue r and gene g, respectively such that,

rf-〖igf〗_(r,g)=〖rf〗_(r,g)×〖igf〗_(r ) (1).

We may view each gene as a vector with one component corresponding to each residue mutation in the gene, together with a weight for each component that is given by (1). Therefore, we can find the overlap score measure with the rf-igf weight of each term in g, for a query q;

Score(q,g)=∑_(r∈q)▒〖 rf-igf〗_(r,g) .

In respect to MRF scoring, this information retrieval method might be applied as follows; the rf-igf weight of a term is the product of its rf weight and its igf weight (W_(r,g)=〖rf〗_(r,g)×log N/〖gf〗_r ) or (W_(r,g)=(1+log⁡〖〖rf〗_(r,g) 〗)×log N/〖gf〗_r ).

That is, firstly, the number of times a residue mutates in a gene (rf=〖rf〗_(r,g)) and secondly, the rarity of the mutation genome-wide in N number of genes (igf=〖N/gf〗_r). Finally, ranking the score of genes for a mutation query q by;

Score(q,g)=∑_(r∈q⋂▒g)▒〖 rf-igf〗_(r,g) .

The score of the query (Score(q,g)) equals the mutations (terms) that appear in both the query and the gene (r∈q⋂▒g). Working out the rf-igf weight for each of those variants (〖rf.igf〗_(r,g)) and then summing them (Σ) to give the score for the specific gene with respect to the query.

Bayesian probability

MRF score may provide a limiting component required for applying Bayesian probability to disease prediction. A clinician may ask for the likelihood of RAG deficiency (or any Mendelian disease of interest) for a patient given a set of gene variants P(H|E) using Bayes’ theorem, P(H│E)=(P(E│H) P(H))/(P(E)) ,

where P(H) is the probability of a patient having RAG deficiency, P(E|H) is the probability of RAG deficiency due to a set of variants that have been pre-emptively assayed, and P(E) is the probability of having a set of gene variants.

P(H) is known since the rate of RAG deficiency is estimated at an incidence of 1:181,000 [68], SCID at a rate of 1:330,000 [2], and we also recently show the rate of RAG deficiency in adults with PID [16]. Being a recessive disease, P(E) must account for biallelic variants and is the most difficult value to determine. This may be found from population genetics data for (i) the rate of two separate, compound heterozygous variants, (ii) the rate of a homozygous variant or potential consanguinity, or (iii) the rate of de novo variation [21]. P(E|H) would be identified where all variants are functionally validated. This requires a major investment, however the MRF score provides a good approximation.
