# Supplementary figures and images for: Predicting the Occurrence of Variants in RAG1 and RAG2
Source: J Clin Immunol. 2019 Aug 6;39(7):688–701. doi: 10.1007/s10875-019-00670-z (PMC6754361; doi:10.1007/s10875-019-00670-z)

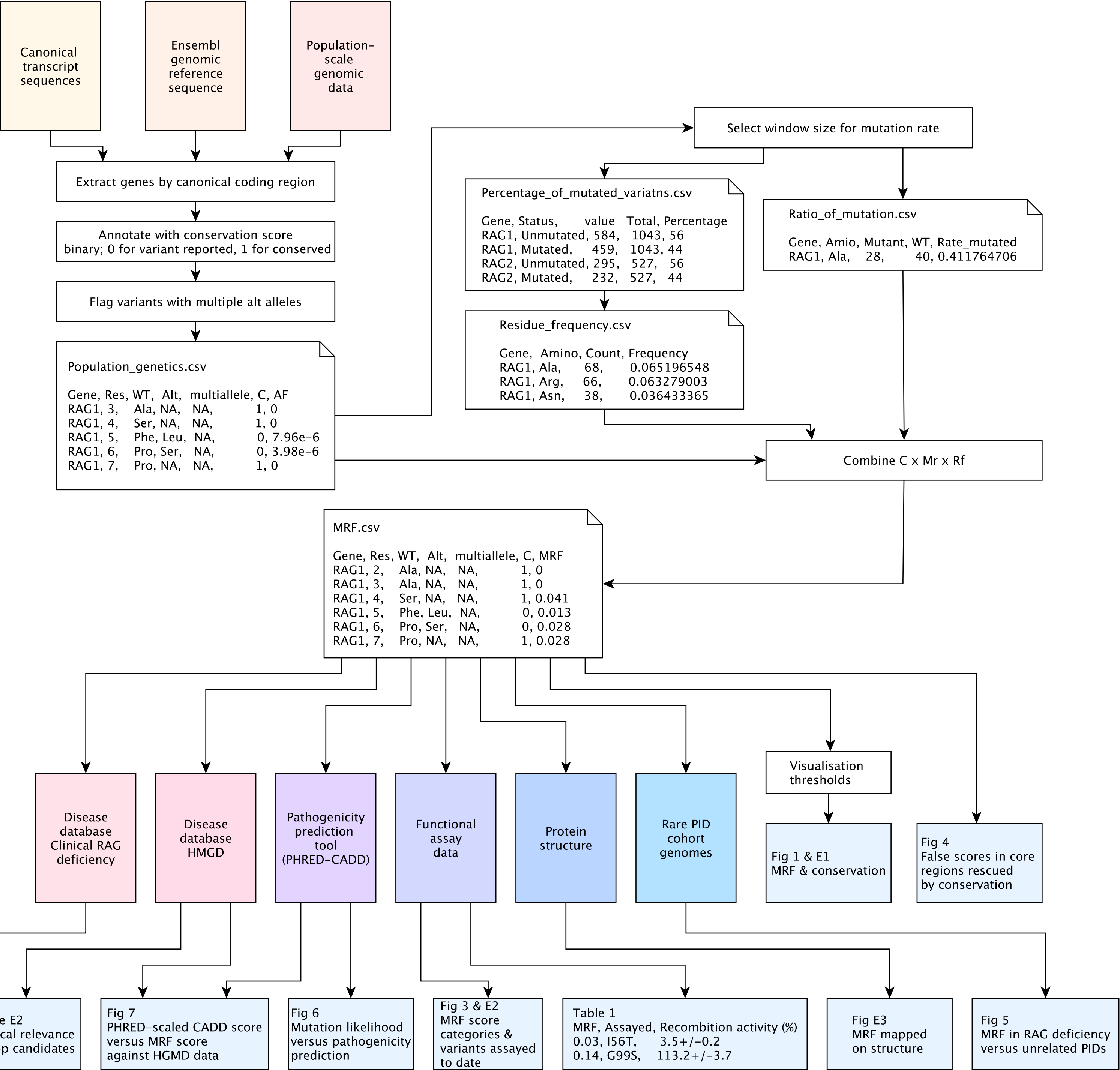

Supplement: Supplementary file 2 — (PDF 259 kb) [file 10875_2019_670_MOESM2_ESM.pdf]

MRF

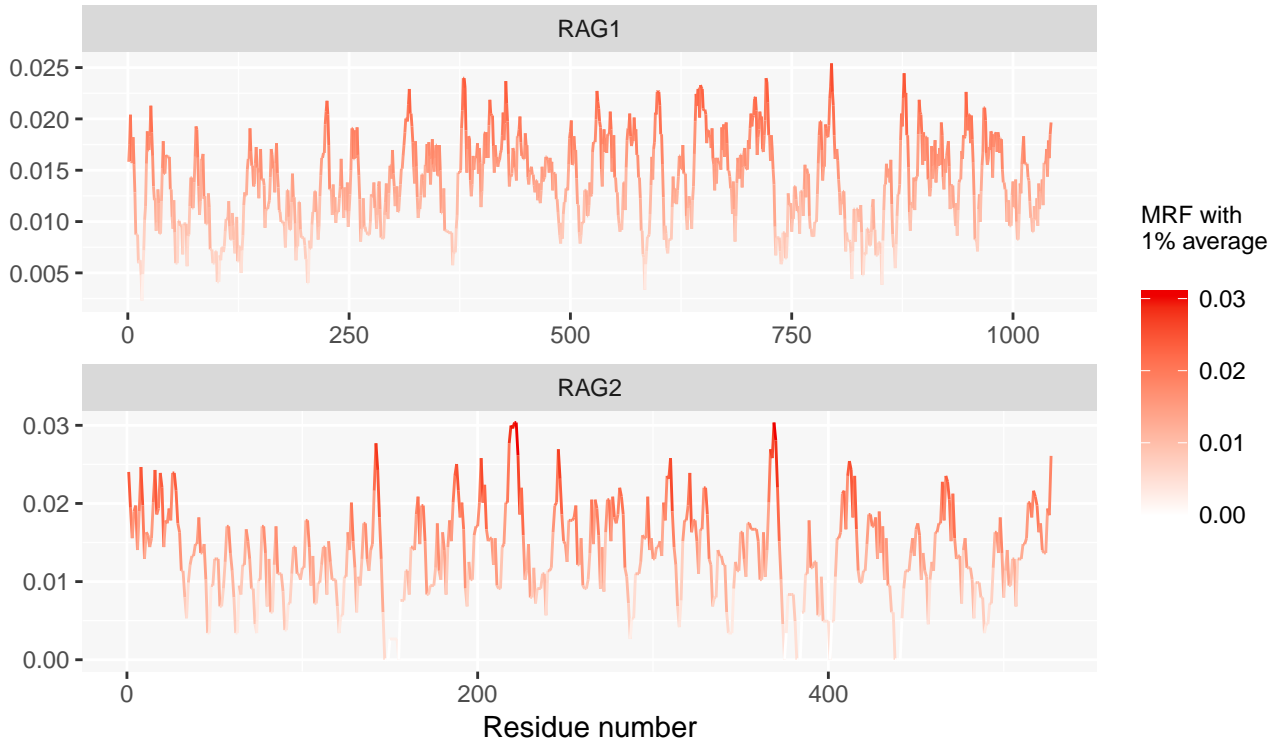

Supplement: Supplementary file 3 — (PDF 18 kb) [file 10875_2019_670_MOESM3_ESM.pdf]

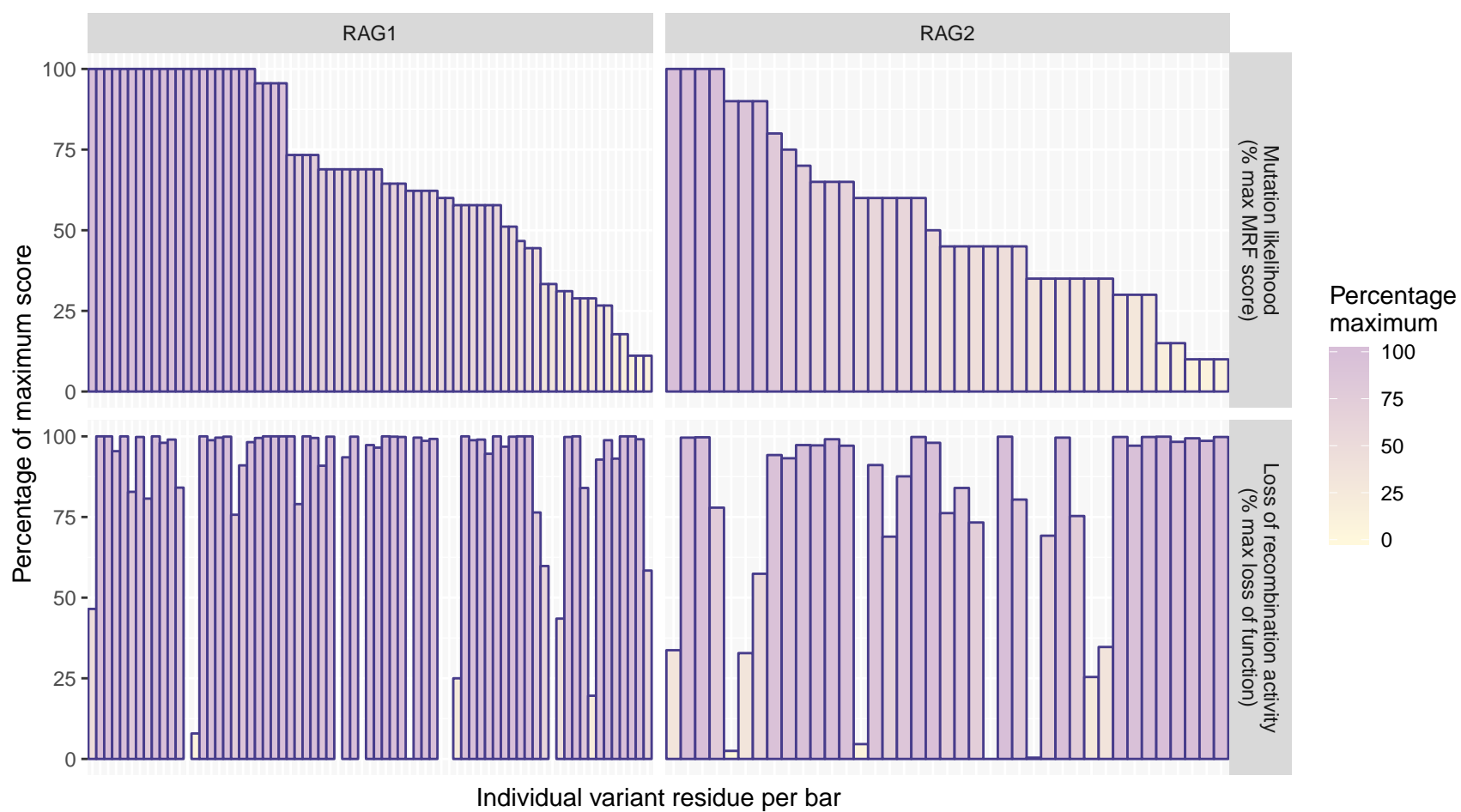

Supplement: Supplementary file 4 — (PDF 8 kb) [file 10875_2019_670_MOESM4_ESM.pdf]

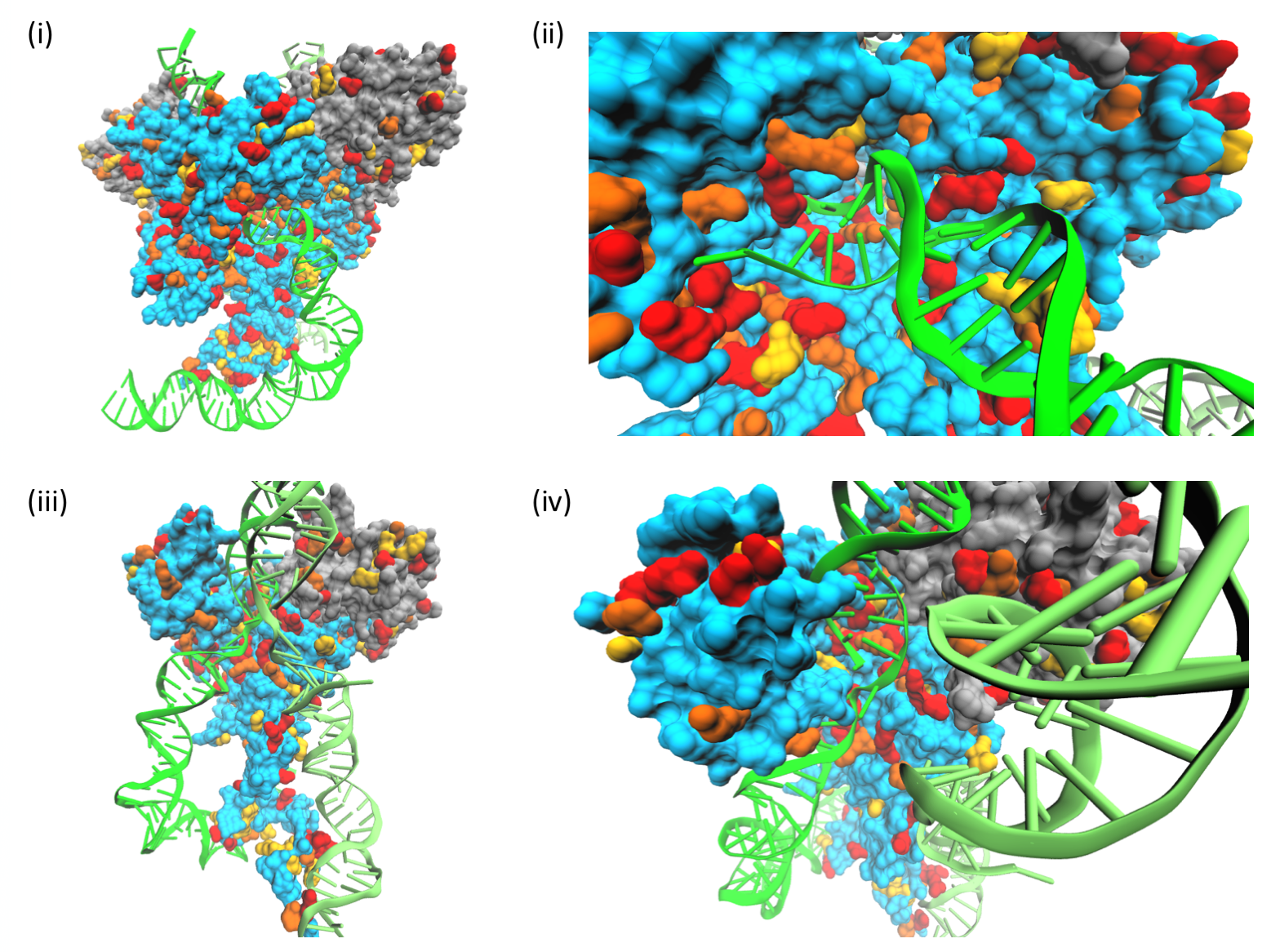

Supplement: Supplementary file 5 — (PNG 1642 kb) [file 10875_2019_670_MOESM5_ESM.png]
